# Supplementary material for: Interstitial Lung Disease Is Associated with Sleep Disorders in Rheumatoid Arthritis Patients
Source: Clocks Sleep. 2023 Dec 4;5(4):755–69. doi: 10.3390/clockssleep5040049 (PMC10742867; doi:10.3390/clockssleep5040049)
Supplement: Supplementary file 1 [file clockssleep-05-00049-s001.zip › clockssleep-2679284-supplementary.pdf]

Supplementary Materials:

Supplementary Table S1: Correlation between sleep quality and clinical characteristics of patients with RA.

Variable	Subjective satisfaction with sleep Pearson p	Insomnia Pearson p	Hypersomnia Pearson p
Clinical characteristics			
Age (years)	-0.439**	0.177	0.375*
Time since diagnosis of RA (months)	-0.174	0.091	0.154
Positive psychological factors			
Resilience	0.106	-0.225*	-0.399*
Emotional intelligence	0.101	0.192	-0.267*
Emotional clarity	0.181	-0.143	-0.334*
Emotional recovery	0.294*	-0.150	-0.381**
Mood disorders			
Anxiety	-0.346**	0.242*	0.243*
Depression	-0.291*	0.277*	0.249*
Inflammatory activity			
DAS28-ESR	-0.261*	0.252*	0.060
Quality of life and physical functioning			
SF-36 PCS	0.190	-0.272*	-0.050
SF-36 MCS	0.100	-0.195	-0.079
HAQ	-0.243*	0.090	0.175
FACIT	-0.018	-0.023	-0.085

**p-value < 0.05; **p-value <0.001.*

Abbreviations. RA: rheumatoid arthritis; DAS28-ESR: 28-joint Disease Activity Score with erythrocyte sedimentation rate; 36-item Short Form Survey (SF-36) PCS: physical component summary; SF-36 MCS: mental component summary; HAQ: Health Assessment Questionnaire; VAS: visual analog scale; FACIT: Functional Assessment of Chronic Illness Therapy – Fatigue scale.

Supplementary Table S2: Univariate and multivariate analyses of the characteristics associated with sleep disorders in RA including treatment.

Predictor	Univariate		Multivariate		
	B	95% CI	B	95% CI	p-value
Sleep satisfaction*					
Female sex	0.308	-0.134, 0.751			
Age > 60 years	-0.771	-1.310, -0.231	-0.624	-1.241, -0.007	0.048
ILD	-0.486	-0.914, -0.058	-0.400	-0.894, -0.032	0.046
Age-CCI	-0.162	-0.288, -0.035			
DAS28-ESR	-0.326	-0.544, -0.109	-0.233	-0.456, -0.016	0.040
Resilience	0.007	-0.001, 0.015			
ER	0.034	0.007, 0.062			
Depression	-0.077	-0.127, -0.027			
Methotrexate	0.138	-0.329, 0.604			
bDMARDs	0.394	-0.138, 0.926			
Mycophenolate	0.246	-1.623, 2.116			

Glucocorticoids	0.061	-0.118, 0.241			
Severity of insomnia**					
Female sex	0.392	-3.101, 3.884			
Age > 60 years	-0.099	-4.544, 4.347			
ILD	6.257	3.148, 9.366	3.953	1.056, 6.851	0.008
Age-CCI	1.240	0.291, 2.430			
DAS28-ESR	2.494	0.797, 4.192			
Resilience	-0.130	-0.184, -0.076	-0.108	-0.160, -0.056	<0.001
ER	-0.138	-0.357, 0.081			
Depression	0.721	0.343, 1.099			
Methotrexate	1.741	-2.355, 5.836			
bDMARDs	0.041	-4.301, 4.383			
Mycophenolate	1.841	-12.951, 11.633			
Glucocorticoids	-0.534	-1.965, 0.896			
Hypersomnia***					
Female sex	0.417	-0.584, 1.417			
Age > 60 years	1.320	0.332, 2.308			
ILD	1.400	0.465, 2.335	1.040	0.086, 1.995	0.033
Age-CCI	0.329	0.043, 0.615			
DAS28-ESR	0.139	-0.379, 0.656			
Resilience	-0.020	-0.037, -0.003			
ER	-0.100	-0.159, -0.042	-0.082	-0.142, -0.021	0.010
Depression	0.103	-0.014, 0.220			
Methotrexate	-0.306	-1.396, 0.784			
bDMARDs	0.135	-1.121, 1.391			
Mycophenolate	-2.277	-6.620, 2.065			
Glucocorticoids	-0.090	-0.504, 0.324			

**Nagelkerke R2 = 0.191; **Nagelkerke R2 = 0.369; ***Nagelkerke R2 = 0.184*

Variables included in the equation: sex, age, diagnosis of ILD, DAS28-ESR, resilience, ER, depression, age-CCI, Methotrexate, bDMARDs, Mycophenolate, Glucocorticoids

Abbreviations. RA: rheumatoid arthritis; ILD: interstitial lung disease; Age-CCI: age-adjusted Charlson Comorbidity Index; DAS28-ESR: 28-joint Disease Activity Score with erythrocyte sedimentation rate; ER: emotional recovery; bDMARDs: biological disease-modifying antirheumatic drugs.

Supplementary Table S3: Classification of the degrees of emotional intelligence according to the 24-item Trait Meta-Mood Scale (TMMS-24).

TMMS variables	Score-Men	Score-Women
Attention	Low ≤ 21	Low ≤ 24
	Adequate 22 to 32	Adequate 25 to 35
	High ≥ 33	High ≥ 36
Clarity	Low ≤ 25	Low ≤ 23
	Adequate 26 to 35	Adequate 24 to 34
	High ≥ 36	High ≥ 35
Repair	Low ≤ 23	Low ≤ 23
	Adequate 24 to 35	Adequate 24 to 34
	High ≥ 36	High ≥ 35

Abbreviation; TMMS: Trait Meta-Mood Scale
